# Collaboration Networks in Applied Conservation Projects across Europe

**DOI:** 10.1371/journal.pone.0164503

**Published:** 2016-10-10

**Authors:** Andreea Nita, Laurentiu Rozylowicz, Steluta Manolache, Cristiana Maria Ciocănea, Iulia Viorica Miu, Viorel Dan Popescu

**Affiliations:** 1 Centre for Environmental Research and Impact Studies, University of Bucharest, Bucharest, Romania; 2 Department of Biological Sciences, Ohio University, Athens, Ohio, United States of America; Tianjin University of Technology, CHINA

## Abstract

The main funding instrument for implementing EU policies on nature conservation and supporting environmental and climate action is the LIFE Nature programme, established by the European Commission in 1992. LIFE Nature projects (>1400 awarded) are applied conservation projects in which partnerships between institutions are critical for successful conservation outcomes, yet little is known about the structure of collaborative networks within and between EU countries. The aim of our study is to understand the nature of collaboration in LIFE Nature projects using a novel application of social network theory at two levels: (1) collaboration between countries, and (2) collaboration within countries using six case studies: Western Europe (United Kingdom and Netherlands), Eastern Europe (Romania and Latvia) and Southern Europe (Greece and Portugal). Using data on 1261 projects financed between 1996 and 2013, we found that Italy was the most successful country not only in terms of awarded number of projects, but also in terms of overall influence being by far the most influent country in the European LIFE Nature network, having the highest eigenvector (0.989) and degree centrality (0.177). Another key player in the network is Netherlands, which ensures a fast communication flow with other network members (closeness—0.318) by staying connected with the most active countries. Although Western European countries have higher centrality scores than most of the Eastern European countries, our results showed that overall there is a lower tendency to create partnerships between different organization categories. Also, the comparisons of the six case studies indicates significant differences in regards to the pattern of creating partnerships, providing valuable information on collaboration on EU nature conservation. This study represents a starting point in predicting the formation of future partnerships within LIFE Nature programme, suggesting ways to improve transnational cooperation and communication.

## Introduction

European Union policies on nature conservation are framed by the Directive on the conservation of natural habitats and of wild fauna and flora, Habitats Directive [1] and the Directive on the conservation of wild birds, Birds Directive [2]. These powerful legal tools standardize nature conservation among EU members [3], an important outcome being the creation of Natura 2000 network, one of the largest ecological network united under a single regulatory framework [4,5]. One of the main objectives of the European Union policies on nature conservation is to safeguard species and habitats of community importance at EU level [6,7]. Notably, the transboundary nature of many conservation issues in the EU requires a close cooperation among member states, at least for improving conservation in transboundary areas [8,9].

A major venue for conservation funding for European priority species and habitats is the LIFE programme, which financed over 1400 projects (over € 1.2 billion) across Europe between 1992 and 2013 [7,8]. The majority of the projects were deemed to be successful, and contributed to achieving the goals of EU conservation policies [8,10,11]. A typical LIFE Nature project targets species and habitats listed by Habitats and Birds Directive, usually within Natura 2000 sites, but there are also projects targeting non-listed species from an EU member state, inside or outside Natura 2000 network [8]. Even though transboundary projects are favored in the evaluation process, only 9% of the LIFE projects have international partners. [10]. Transboundary cooperation is achieved mostly through partnership (e.g., a project is implemented in one country, but with partners from multiple countries), but information regarding the degree of cooperation among EU partners is lacking. Thus, there is a need to understand the extent of transboundary partnerships, because a higher cooperation between multi-national partners can facilitate the transfer of knowledge and best practices leading to successful implementation of policies across multiple member states [12,13]. LIFE projects are also an important venue for fostering collaboration across sectors and between different types of institutions. LIFE projects have been implemented by a large number of public agencies, protected areas administrations, NGOs, as well as research and education institutions [8,10]. The diversity of project beneficiaries and their partners highlights the fact that implementation of Habitats and Birds Directive relies heavily on collaboration between stakeholders and environmental authorities. Several studies confirmed the institutional diversity in this area, and the diversity of partnership structures. In some Member States the top-down approach is favored (e.g., Romania) while in others bottom-up approach is present (e.g., Netherlands) [9,14–17]. Bottom-up approach could be more productive, flexible and capable to solve conflicts, while a top-down approach can lead to a faster implementation of EU biodiversity policies [14,18].

Exploring the links between partners in LIFE projects is key to evaluate how collaborative efforts are evolving in EU applied conservation projects [19], and to identify key players in European conservation. Social network theory is a widely used tool to evaluate the importance, distribution and structure of links between partners [20,21]. In this case, social network analysis can help map the likely complex networks resulting from partnerships emerging from more than two decades of LIFE Nature funding, which could promote our understanding on dynamics of large-scale geographic networks [22,23]. Social network analysis provides a variety of metrics by which partnerships can be evaluated (see Glossary in S1 Table) [21]. For example, centrality of a network member is a fundamental concept that identifies which nodes (e.g., partners) are more “central” than others, thus influential in European or country-specific conservation efforts [24]. Metrics such as this, quantified for the LIFE Nature partners network, can answer critical questions of which country, institutions, and what type of organizations are most influential, but also which are key actors in EU applied conservation and the LIFE Nature network as a whole (both across national and EU levels).

More specifically, the social network analyses applied to our study highlight collaboration formed by two sets of nodes: (1) one set representing the project (as the collaboration venue) and (2) one set representing the projects partners. Such an approach has been used successfully for analyzing the structure of collaboration in various fields, such as water management, urban development, conservation initiatives [13,25,26]. The analysis of a collaborative network can reveal not only the most influent players, but also which players have the potential to control communication between different partners, and which partners are independent from such control or efficient in approaching key players [27–29]. Project implementation, cooperative behavior or collaborative approaches are increasingly used to address challenging environmental problems worldwide [30,31]. While “bottom-up” approach focuses on stakeholders developing and implementing projects, the “top-down” approach is based on higher level governments directing collaborative efforts and project implementation [32].

The aim of our study is to understand the capacity of EU member states to work together for implementing the Birds and Habitats Directives in the framework of the LIFE Nature programme, and to identify the structure of collaborative networks in different EU countries. Specifically, using social network theory, we asked the following questions: (1) which countries are most influential in LIFE Nature partnership network as a whole; (2) which organizations are most important in implementing LIFE projects at country level; and (3) why and how partnership arise in LIFE projects, with particular emphasis on characterizing the different organizational categories involved in LIFE Nature projects. We implemented two sets of analyses: (a) an European-level analysis, in which we assessed the network resulted from all LIFE projects awarded between 1996–2013 (centrality of EU countries), and (b) a country-level analyses (centrality of LIFE Nature projects beneficiaries and within-country structure of partnerships), in which we selected the LIFE projects implemented in six member states with different policy background and approaches to collaboration. These were: United Kingdom and Netherlands, where a bottom-up approach to conservation is well developed; Greece and Portugal, characterized by a mix of top-down and bottom-up approaches; Romania and Latvia, where the top-down approach prevails and the bottom-up approach is at an exploratory stage [14,15,18,33,34].

## Methods

### Network data

The data used in this study were obtained from LIFE database [10]. The database includes all the LIFE projects financed by European Commission starting with 1992. Specifically, we analyzed the projects financed between 1996 and 2013 in three out of the five LIFE Nature funding schemes: LIFE II (1996–1999), LIFE III (2000–2006), and LIFE+ (2007–2013) work programmes. We excluded LIFE I (1992–1996) from our analysis, as these projects were awarded to a single beneficiary (i.e., there were no officially recognized partners), as well as LIFE 2014–2020 due to the fact that the call is ongoing.

For each project we extracted the project ID, coordinating beneficiary, associated beneficiaries, project duration, country of coordinating beneficiary, target countries, project title, and year financed. We standardized the name of the coordinating and associated beneficiaries (hereafter *organizations* or *partners*) in order to avoid including the same organization as two or more independent bodies. We then classified the organizations as (1) public authorities, (2) protected area administrators (i.e., organizations mostly dedicated to PA management), (3) NGOs, (4) research and education bodies, and (5) enterprises (i.e., industry, private businesses, state owned businesses).

To analyze the data by using social network theory methods, we constructed bipartite networks, were organizations are tied together if they participate in the same LIFE Nature projects. In bipartite networks ties are formed between the two sets of nodes (two-modes), i.e., *n* organizations (set A) and *m* LIFE Nature projects (set P). The networks were stored as a *n* by *m* matrix, where a link between organization *i* and LIFE Nature project *j* takes value 1 if present and 0 otherwise [35].

### Transboundary cooperation—centrality of EU countries

To analyze the centrality of EU countries, we replaced each organization participating in a project with the country of origin of that particular organization. By using the newly designed matrix, we calculated four network metrics: degree centrality, betweenness centrality, closeness centrality, and eigenvector centrality. The metrics were calculated using two-mode data algorithms implemented by UCINET software 6.611 [36].

Degree centrality represents the number of ties a node has, divided by the maximum number of possible ties in the respective set (i.e., organizations, projects). Betweenness centrality refers to the number of shortest paths that pass through a node, normalized by the maximum betweenness that any node can achieve. A country with high betweenness might play an important role in the network because it mediates the interaction between the linked nodes. Eigenvector centrality of a node is the principal eigenvector of the adjacency matrix defining the network, normalized by dividing the raw eigenvector score by maximum score attainable in the respective set, thus being determined by the number and influence of its neighbours [37]. Because the eigenvector score of a node is determined by the eigenvector scores of adjacent nodes, the measure can be interpreted as a measure of the influence of a node. For example, a country with high eigenvector score is likely connected with other high scoring organizations [20,38]. Closeness centrality of a node is inversely proportional to the total geodesic distance to all other nodes in the respective set. Nodes in the middle of a large network has a greater influence in the network, as are close to many other nodes and can facilitate the information flow [21,38].

### Within-country cooperation–centrality of LIFE Nature projects beneficiaries

To analyze country-specific network properties, we selected six EU member states as case studies: United Kingdom and Netherlands as representatives for Western Europe, Greece, and Portugal as representatives for Southern Europe, and Romania and Latvia as representatives for Eastern Europe (also new EU members, since 2007 and 2004, respectively). For United Kingdom we found 120 distinct organizations participating in 46 projects, for Netherlands 54 organizations in 40 projects, for Greece 124 in 57 projects, for Portugal 156 organizations in 63 projects, for Romania 119 organizations in 48 projects, and for Latvia 122 organizations in 28 projects (see S1 Dataset).

To characterize the network and analyze the relative structural importance of organizations, we used also standard cohesion and centrality metrics for two-mode data [36]. Specifically, we used network density, fragmentation, and average geodesic distance as cohesion measures, and degree centrality, betweenness centrality, and eigenvector centrality as measures of organizations centrality measures [21,38].

Network density represents the number of ties divided by *n* × *m*, hence, indicates what proportion of all possible ties between organizations and projects are actually present in the observed network. A higher density indicates a network with high proportion of connections between partners. The average geodesic distance is the average geodesic path length between a pair of nodes (short-path), within networks components. A lower average geodesic distance indicates a higher cohesion. Fragmentation is zero when no isolated projects are present, thus, all network nodes are connected [21,38]. Interpretation of degree centrality, betweenness centrality, and eigenvector centrality is similar with the one from analysis of centrality of EU countries, but reflects the organizations within a country.

### Within-country structure of partnerships

We used ERGM (p* models) to understand how and why network links arise [35,39] in the six countries selected as case studies. To analyze the contribution of each category of organization to network structure we first allocated each organization an organizational type (public authority, protected area administrator, NGO, research and education body, and enterprise), and then coded the type as dummy values: 1 –the attribute is present, 0 –the attribute is not present. The resulted matrices were analyzed with BP-Net, an extension of P-Net for bipartite networks [35,40].

ERGMs assign probability to selected graphs according to configurations statistics using the following generalized form [40]:

$$P_{\theta }(X=x)=\frac{1}{k(\theta )}exp\sum_{q}\theta_{q}z_{q}(x)$$

where, *q* are network configurations, *θ* is a set of parameters with: *θ*_*q*_ parameter for configuration *q*, *z*_*q*_*(x)* graph statistic for configuration *q* (which weights the relative importance of the respective configuration), and *k(θ)* normalizing constant to force the probability of all graphs to add to 1.

ERGMs for bipartite networks can accommodate several structural within-node set configurations (e.g., density, stars of different sizes, alternating stars, edge cycles) and between set configurations for (e.g., activity, across-type bridging, within-type bridging) [35,39,40].

To fit an ERGM, we first selected the network configurations to be estimated, run the model with BP-Net default values, and checked the model statistics. If *t*-ratios for selected network configurations were <4 for all values, we updated the parameters with the new estimated values, and re-run the model using an increased multiplication-factor. We repeated the procedure until the model converged or we selected other configurations if the model was unreliable. A model was considered as converged only if all selected parameters had *t*-statistics <0.1 in absolute values [41]. Finally, the converged models were analyzed using a Goodness-of-Fit procedure using 100,000,000 simulations [42]. A model with a good fit has the absolute value of the *t*-ratios <0.1 for converged parameters, and <2 for other network statistics [41]. We reported the Mahalanobis distance as an overall fit of the model [42]. A model with a small Mahalanobis distance captures well the observed network. All the models were fitted with fixed density of the observed network.

To test whether the formation of ties was a result of organization types we included in the converged models the following networks configurations: organizations types activity (RA), across-type bridging for organizations (TsoA1), within-type bridging for organizations (TsoA2) [40,41]. S2 Table presents the description of graph configurations in ERGM and interpretation of tested binary attribute effects.

The methodology applied in this study is synthetized in S1 Fig. Networks graphs were represented using NodeXL (https://nodexl.codeplex.com/).

## Results

### Transboundary cooperation—centrality of EU countries

The LIFE Nature program funded 1261 projects between 1996 and 2013. The average number of projects awarded per country was 40.67 (median = 34; range = 5–217). The most successful countries in terms of number of awarded project are Italy (217 projects) and Spain (175 projects). The least active countries are Malta (5 projects), Czech Republic (6 projects), Cyprus (7 projects), and Luxembourg (8 projects). The average number of partners per project for all countries was 3.56 (median = 3; range = 1–22). The majority of partners were public authorities (45.91%) and NGOs (25.56%), followed by research and education institutions (11.56%), protected area administrators (10.31%), and enterprises (6.66%) (see S1 Dataset).

**Fig 1:**
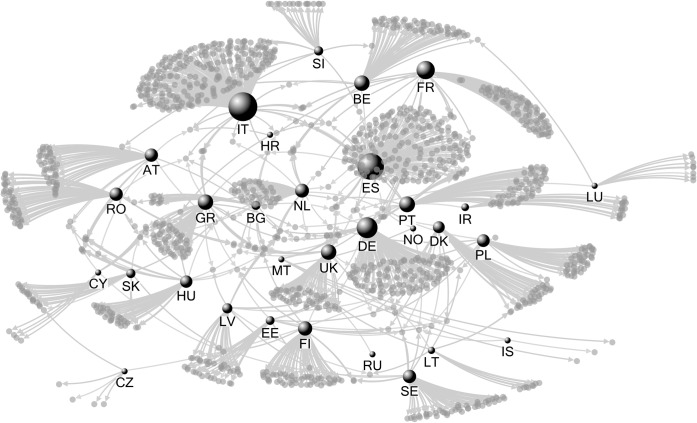
Country-level LIFE projects partnership network.

When analysing the ties between countries (e.g., transboundary partnerships in LIFE Nature projects), Italy plays the most important role among all the EU member states, having the highest degree centrality (0.177), eigenvector centrality (0.989) and betweenness centrality (0.329) (Fig 1, S3 Table). Hence, Italian partners have links with important organisations from other countries and can play a significant role in knowledge transfer and communication. Simultaneously, Spain records high values of some centrality metrics (degree = 0.149, betweenness *=* 0.278), but a low eigenvector value (eigenvector = 0.129). Partners from Netherlands have a low degree (0.041), yet the closeness index has the highest value among all countries (0.318), thus this country can communicate fastest within the network, being close to many countries in terms of geodesic distance. Several other countries have a high closeness such as: Spain (0.316), Germany (0.313), Italy, and France (0.299). By contrast, Greece has high betweenness, but low closeness, suggesting that Greek organisations share ties with a smaller number of countries than the rest of the network (Fig 1).

### Within-country cooperation–centrality of LIFE Nature beneficiaries

**Fig 2:**
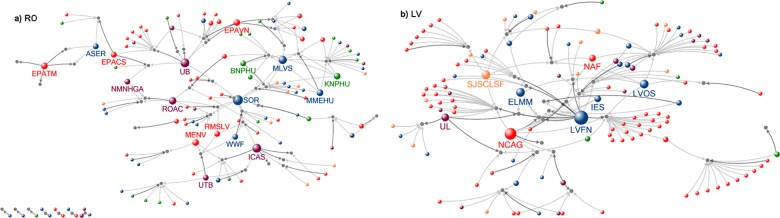
LIFE Nature partnership network for Romania and Latvia (size of nodes for organisation = degree; circles: red—public authority, blue–NGO, green—park reserve authority, purple—research and education, orange–enterprises, grey–project; dark grey arrow—link to a beneficiary, grey arrow—link to a partner; isolated projects are presented in the left bottom of a network; abbreviations are shown in S4 Table).

LIFE Nature networks level indicate low density networks for all analyzed countries (Fig 2, S2 Fig, Table 1). With the exception of Latvia, the other five countries considered here have projects that do not have links to the main component of the network (isolated projects; i.e., single organizations or groups of organizations that have been awarded single projects, and have not formed partnerships with any other beneficiary or associated organizations in other projects).

**Table 1:** Cohesion metrics for six LIFE Nature partnership networks.

Country	Density	Average distance	Network diameter	Fragmentation
**United Kingdom**	0.039	4.195	8	0.200
**Netherlands**	0.047	3.229	6	0.517
**Greece**	0.028	5.654	12	0.255
**Portugal**	0.023	4.893	10	0.389
**Romania**	0.034	5.965	15	0.214
**Latvia**	0.051	4.662	10	0.000

**Fig 3:**
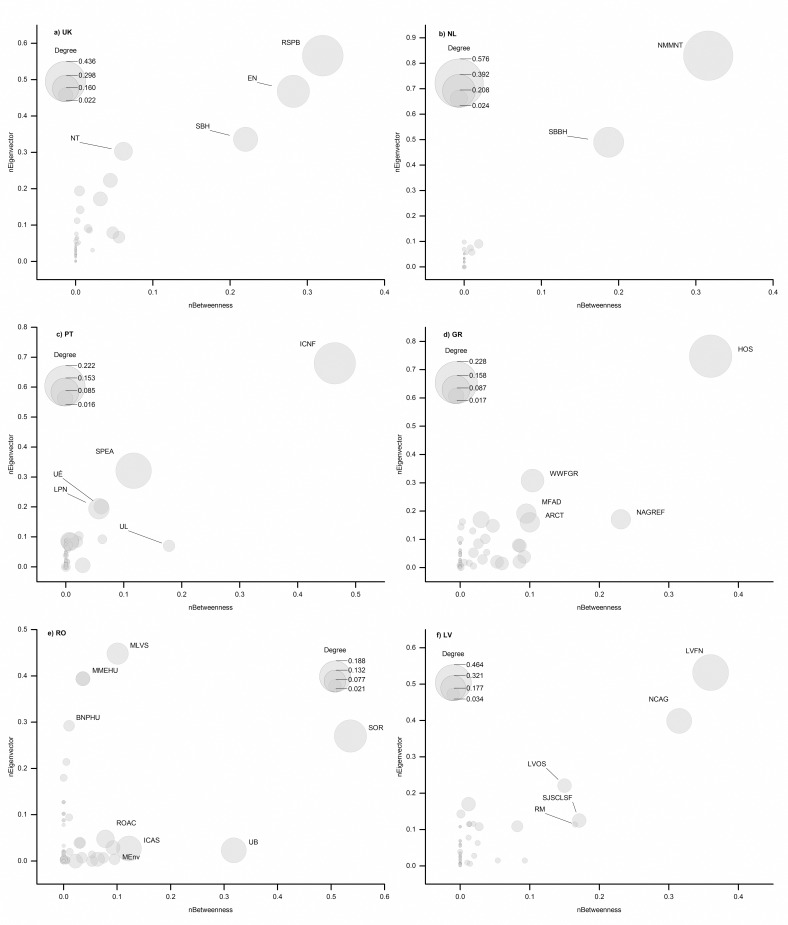
Betweenness vs. Eigenvector centrality for United Kingdom, Netherlands, Portugal, Greece, Romania and Latvia (abbreviations are shown in S4 Table).

In the UK (Fig 3A), the Royal Society for the Protection of Birds (NGO) dominates the network, having the highest degree, eigenvector, and betweenness metrics. RSBP is followed by two public authorities: English Nature and Scottish Natural Heritage in terms of their importance in the UK LIFE Nature network. In Portugal, the network of LIFE Nature projects is centered on ICNF (Instituto da Conservação da Natureza e das Florestas) (Fig 3C). A similar centralized network characterizes Netherlands, where Natuurmonumenten (NGO), and the public authority Staatbosbeheer are the most active organizations in the LIFE Nature programme (Fig 3B). For Greece (Fig 3D), Hellenic Ornithological Society (NGO) has the most connections, while for Latvia, Latvian Fund for Nature (NGO) and Nature Conservation Agency (public authority) are most successful organizations in attracting LIFE funding (Fig 3F). In contrast to these five countries, the Romanian network (Fig 3E) shows organizations that have high values for the eigenvector but low value of betweenness index (e.g., NGOs Milvus Group Association and MME / BirdLife Hungary), as well as institutions that have a high betweenness value, but low eigenvector value, such as Romanian Ornithological Society (NGO) and University of Bucharest.

### Within-country structure of partnerships

ERGMs with structural effects converged for all analyzed countries and fitted the observed networks well (see S2 Dataset). In all six networks, the propensity for organizations to create ties is higher than expected for 2^nd^ order degree distribution effects (2-stars estimates positive), but lower than expected for higher orders (3-stars, k-stars). Thus, very popular projects and organizations have a lower than expected probability of receiving further ties (Table 2).

**Table 2:** Structural ERGMs for selected LIFE Nature partnership network.

Parameter	UK	NL	PT	GR	RO	LV
**2-star A**	3.06 (0.61)*	0.49 (0.06)*	12.00 (1.93)*	2.65 (0.41)*	7.27 (1.04)*	3.30 (0.47)*
**2-star P**	3.19 (0.74)*	0.40 (0.10)*	5.97 (1.52)*	8.96 (1.03)*	4.17 (1.14)*	0.23 (0.04)*
**3-star A**	-0.10 (0.02)*	-0.02 (0.01)*	-0.31 (0.05)*	-0.30 (0.07)*	-0.98 (0.21)*	-0.39 (0.08)*
**3-star P**	-0.02 (0.01)*	-0.02 (0.02)	-0.05 (0.01)*	-0.09 (0.06)	-0.07 (0.04)*	-0.01 (0.01)*
**L3**	-0.03 (0.00)*	-0.03 (0.01)*	-0.03 (0.01)*	-0.05 (0.02)*	-0.06 (0.01)*	-0.02 (0.01)*
**C4**	-1.19 (0.41)*	-	-5.52 (1.09)*	-3.52 (0.62)*	-1.22 (0.59)*	0.14 (0.07)*
**ECA**	-	-	-	-0.10 (0.05)	-	-
**Ksa**	-3.96 (0.52)*	-	-6.25 (0.71)*	-5.33 (0.78)*	-9.38 (1.48)*	-6.92 (1.01)*
**Ksp**	-	-	-1.52 (0.48)*	-	-2.20 (0.93)*	-
**Kca**	-1.59 (0.58)*	-	-9.26 (1.94)*	-	-1.75 (0.61)*	-
**Kcp**	-2.88 (0.74)*	-	-5.34 (1.51)*	-8.65 (1.01)*	-3.40 (1.09)*	-
**AECA**	-	-	-	0.39 (0.15)*	-	-
**Mahalanobis distance**	6.58	8.04	6.53	3.05	5.98	3.40

* An asterisk denotes statistically significant estimate at alfa = 0.05.

See S2 Dataset for full results.

United Kingdom, Portugal, and Romania had lower than expected number of conservation hubs and tendency for organizations to participate in popular projects (e.g., the closure effect—potential closures -L3 and full closures–C4—are underrepresented). In Greece, there is the tendency to collaborate in a second project with partnered organizations, given the fact that the full-closure effect is positive which suggest a slight tendency for close-knit partnerships (Table 2).

The tendency for organizations to be involved in multiple projects (KCA) is negative for United Kingdom, Portugal, and Romania. The same negative relation holds for the tendency for projects to share multiple organizations (KCP) for United Kingdom, Portugal, Greece, and Romania. In Greece, very active organizations are brokers between closed partnership and the rest of the network, and thus, help in sharing information (AECA positive) (Table 2).

The activity level (RA) of each organization category (public authority, park authority, NGO, research and education, enterprises) can be explained by dyadic dependent ERGMs for United Kingdom, Portugal, Greece, Romania, and Latvia (Table 3). We found that no organization category acts as ‘across-type bridging’ organization in the analyzed networks (e.g., negative tendency for a category of organizations to connect to projects implemented by different categories; TSoA1). However, there are more than expected cases of organization categories that are ‘within-type bridging’ (e.g., they tend to connect to the same organization categories within projects): public authorities in Netherlands and Latvia, NGOs in Netherlands, Portugal, Greece, and Latvia, research and education entities in Netherlands, Portugal, Romania and Latvia, enterprises in Portugal and Latvia, and park authorities in Romania (TSoA2) (Table 3).

**Table 3:** Within–node ERGMs for selected LIFE Nature partnership networks.

Parameter	UK	NL	PT	GR	RO	LV
**Activity (RA)**						
**Public authority**	-1.27 (0.20)*	-	1.07 (0.25)*	0.92 (0.25)*	-1.21 (1.20)*	-1.75 (0.29)*
**Park authority**	-2.22 (0.57)*	-	1.66 (0.17)*	0.09 (0.59)	-1.32 (0.30)*	-1.13 (0.23)*
**NGO**	-1.34 (0.20)*	-	1.53 (0.16)*	1.61 (0.23)*	-1.06 (0.24)*	-1.00 (0.14)*
**Research and education**	-2.01 (0.39)*	-	1.52 (0.17)*	1.52 (0.24)*	-0.79 (0.26)*	-1.18 (0.19)*
**Enterprises**	-1.41 (0.31)*	-	0.64 (0.45)	0.74 (0.46)	-2.65 (0.83)*	-1.22 (0.21)*
**Mahalanobis distance**	6.66	-	6.68	3.47	5.98	3.89
**Across-type bridging (TSoA1)**						
**Public authority**	-0.09 (0.06)	-	-0.19 (0.04)*	-0.23 (0.11)*	-0.17 (0.07)*	-0.20 (0.03)*
**Park authority**	-0.13 (0.08)	-	-0.09 (0.04)*	-0.13 (0.13)	-0.08 (0.06)	-0.13 (0.06)*
**NGO**	-0.12 (0.04)*	-	-0.17 (0.04)*	-0.02 (0.11)	-0.10 (0.08)	-0.11 (0.03)*
**Research and education**	-0.13 (0.05)*	-	-0.15 (0.04)*	-0.05 (0.10)	-0.05 (0.06)	-0.09 (0.03)*
**Enterprises**	-0.05 (0.03)	-	-0.11 (0.04)*	-0.18 (0.12)	-0.10 (0.07)	-0.13 (0.04)*
**Mahalanobis distance**	6.82	-	6.56	3.12	5.98	3.58
**Within-type bridging (TSoA2)**						
**Public authority**	-	0.17 (0.08)*	-0.01 (0.10)	-0.16 (0.14)	0.04 (0.08)	0.09 (0.02)*
**Park authority**	-	-	0.03 (0.54)	-	0.18 (0.05)*	-
**NGO**	-	0.23 (0.11)*	0.11 (0.04)*	0.26 (0.09)*	0.09 (0.07)	0.22 (0.03)*
**Research and education**	-	0.64 (0.12)*	0.19 (0.06)*	0.14 (0.22)	0.25 (0.11)*	0.37 (0.07)*
**Enterprises**	-	-	0.37 (0.04)*	-	0.32 (0.21)	0.30 (0.14)*
**Mahalanobis distance**	-	7.12	6.37	4.14	6.18	3.44

Estimate and standard error.

* An asterisk denotes statistically significant estimate at alfa = 0.05.

See S2 Dataset for full results.

## Discussion

Our social network analyses highlighted the structure of collaboration in the most prominent conservation funding scheme in the European Union–the LIFE Nature programme, which may be considered as network of networks (e.g., multilayer networks) [43]. By using LIFE Nature projects and beneficiaries as network data, we described the patterns of transnational and within-country cooperation and highlighted the key players (both as organizations and countries) in nature conservation in the European Union.

### Collaboration between EU countries

Collaboration in nature conservation can take many forms [44,45], and the EU’s LIFE Nature programme became one of the mainstream venues for collaboration towards the implementation of Habitats and Birds Directives at member-state- and EU-levels [3,8,11]. In this policy field, given that in Europe state boundaries or organizational foci are less relevant for achieving EU conservation objectives [46], we expected a high level of cooperation across- and within-countries. Yet, the majority (91%) of the LIFE projects have no international partners (Fig 1), suggesting that despite the continental approach of EU’s environmental policies, conservation is still mostly performed on a country-by-country basis [4,11].

However, transboundary collaboration does exist within the LIFE Nature projects, as highlighted by the network centrality metrics. In LIFE Nature programme, Italy, Spain, Germany, and France have the highest degree centrality and betweenness metrics, suggesting that these countries are successful in attracting LIFE Nature funding (i.e., largest number of projects awarded), and that they have the most influence over the information flow (i.e., other countries must go through to achieve communication with the network members [38], which from the temporal perspective could be defined as organization to organization communication, either one to many information dissemination [47]). These results are not that surprising; for example, Italian beneficiaries collaborate with 10 different countries (Belgium, Bulgaria, Croatia, France, Greece, Netherlands, Portugal, Romania, Slovenia and Spain) in 11 projects awarded to Italy, as well as in six other projects awarded to France, Greece, and Slovenia. In contrast, although UK beneficiaries work in a larger number of projects outside of their country (18 projects from 10 countries, and one project awarded to UK but implemented in Romania), UK has a lower betweenness value, and hence, a lower importance in transnational information flow. This can be explained by the fact that most of the UK transnational projects include >2 countries, including the key players (Italy, Spain, Germany, France). For example, the UK project LIFE04 NAT/GB/000245 has partners from 9 countries [10] and thus, the information can flow through other countries than UK.

In terms of overall influence (e.g., a country that is most likely to be part of different partnerships with other influent countries), emphasized by the high eigenvector centrality [27], Italy is by far the most influent country in the European LIFE Nature network. In contrast, Spain and Germany have considerably lower eigenvector scores, and thus collaborate with “remote” countries (at the periphery of the network).

The closeness index showed a very different situation. The country with the highest value is Netherlands, followed by Spain and Germany, suggesting that these can communicate fastest with other network members and thus, these states are able to efficiently promote knowledge sharing with most of EU members states [48]. The contrast between low betweenness and high closeness ranking of Netherlands can be explained by connections with partners from countries with many projects (e.g., Germany, Italy, and Spain), which control the flow of information between EU countries.

Overall, Eastern European countries have lower centrality scores than most of the Western European countries (see S3 Table), which could be partially explained by the time lag in accessing LIFE Nature funding in Eastern Europe. For example, Romania, the seventh largest country in EU, gained access to the LIFE funding scheme in 1999 (seven-year time lag), was awarded as many projects as the UK, but it is still peripheral in terms of cooperation and influence in the European network. Other large Eastern European countries, such as Poland and Hungary are in similar situations, suggesting that transboundary cooperation should be emphasized and better incorporated in the LIFE Nature calls by the European Commission, including guidance for stimulating cooperation between Eastern and Western European countries (e.g., presenting case studies of transboundary cooperation).

### Within-country partnerships

In all but one country (Romania) our network analyses showed that key players are both important and influent (Fig 3). These networks are dominated by a few key players: UK–RSPB (NGO), English Nature (public authority), and Scottish Natural Heritage (public authority); Netherlands–Natuurmonumenten (NGO) and Staatbosbeheer (public authority); Portugal–Instituto da Conservação da Natureza e das Florestas (park authority); GR–Hellenic Ornithological Society (NGO); Latvia–Latvian Fund for Nature (NGO) and Nature Conservation Agency (public authority). The network created around RSPB, and therefore the level of transnational cooperation is much higher if taking into account all the branches of this organization in other countries (e.g., Romanian Ornithological Society, Bulgarian Society for the Protection of Birds, MME BirdLife Hungary, BirdLife Italy, Sociedad Española de Ornitología, etc.). RSPB is an NGO focused on nature conservation in general (http://www.rspb.org.uk/), not only birds, and was involved in the implementation of 28 LIFE Nature projects, from which 10 were focused mainly on habitats, biodiversity issues, and climate change adaptation (S1 Dataset).

While we were expecting a higher number of NGOs involved in LIFE projects in the Western and Southern Europe compared to Eastern Europe due to the longer and more established NGO-government relations from the Western Europe [16,49,50], the current situation is more complex (Fig 3). Across all six analyzed case studies, Romania’s network is the most intricate (Fig 3E). Here, there are organizations with high betweenness (e.g., SOR/BirdLife—NGO, Bucharest University and Forest Research Institute—research and education), while others have high eigenvector values (e.g., Milvus Association and MME / BirdLife Hungary—NGO). The cooperation of the Milvus Association with Hungarian organizations influenced the network structure, suggesting that the Romanian network is a case of good practice in transnational cooperation in applied conservation. This structure was likely favoured by the complex Romanian protected area governance system, which engages a plethora of local or national organisations, including public authorities and NGOs [51].

Further, structural ERGMs provided insight into the network structure via cohesion and centrality metrics [40,52]. The positive 2-star effects for projects and organizations confirm a centralization in the network, since there are several active organizations (i.e., partners in many projects) and several popular projects (i.e., projects with many partners) in all six networks. However, negative estimates for higher degree star effects, such as 3-star and alternating k-stars, suggests a limit beyond the number of projects awarded to an organizations and the number of partners in projects (median = 3). In the UK, Portugal, and Romania, organizations awarded a project are less prone to participate in other projects; thus, the accumulated advantage effect of knowledge transfer to other organizations or projects is minor [52]. Such limited cooperation can be attributed to the difficulty of implementing LIFE Nature projects, as well as the fixed budged per country, which caps the total number of projects awarded to a country in a given multiannual programme [8]. Because of the challenges posed by LIFE projects [11], many organizations prefer to finish a project before applying for another. A typical LIFE Nature project unfolds over > 4 years, and the accumulation of experience (e.g., coordination, reporting, implementation) can be a slow process. While some organizations are clearly very successful in terms of number of attracted projects (e.g., RSBP), their experience may not extensively shared or easily accessible to a larger number of organizations. Such organizations do not tend to participate in popular projects (with many partners). However, in cases where cooperation between a leader and a local organization emerge, LIFE projects have the potential to unlock a governance model based on participatory management [18].

The level of activity for the analyzed organization categories (public authorities, protected areas authorities, NGOs, research and education, and enterprises) is either lower (UK, Romania, Latvia) or higher (Portugal, Greece) than expected in the respective country (Table 3), which suggests that all categories are equally successful in attracting LIFE Nature projects. This result was unexpected because we hypothesized that participation of public authorities in Eastern European countries would be greater compared to the Western European countries [14]. Thus, our results suggest that applied conservation is performed equally by all organization categories, which is beneficial for biodiversity and sustainable development [53]. Despite these benefits, ERGM results highlight a limited potential for learning from other organization types. In our networks the ‘across-type bridging’ estimates were negative or not statistically significant, suggesting a lower tendency to cooperate with other organization types. This can be interpreted as a tendency to avoid risks [26], and likely an artifact of different norms governing each organization types [45]. On contrary, ‘within-type bridging’ was higher than expected. Public authorities tend to disproportionally link to projects including public authorities in the Netherlands and Latvia. Same tendency is valid for NGOs in Netherlands, Portugal, Greece and Latvia, research institutions in Netherlands, Portugal, Romania, and Latvia, and enterprises in Portugal and Latvia. These organizations display a greater than expected close partnership with similar types of organization, which can be also interpreted as the tendency to avoid cooperation with other organization types [40,54]. However, for Romania, park authorities have greater than expected organizational homophily, which can be interpreted as a sign of cooperation among protected areas in LIFE Nature projects.

## Conclusions

While our approach provides valuable information on collaboration around nature conservation in the EU, many network processes (such an information flow) are theoretic [44]. Thus, the outcome can be used as a starting point for country by country analyses targeting e.g., diffusion of innovation in LIFE projects, predicting partnership performance, temporal development of the networks, geographic constraints [21,55,56]. This information can be further used by policy makers and scientists to provide benchmarks for collaboration potential for the LIFE programme. Currently, project evaluations within the LIFE programme are performed by analyzing the project outcomes, resulting in an improvement of guidelines for applicants and adjustments of overall objectives [57]; these evaluations do not contain provisions for stimulating of within-country and within-EU collaboration [7]. Although the European Commissions shall ensure a proportional distribution of funds among all Member States based on population and number of Natura 2000 sites, transnational partnerships and also, those containing many types of organizations should be stronger encouraged, in this way ensuring the information flow and gained experience across the entire LIFE Nature network. Our research suggests that promoting cross-country collaboration may close the gap between the Eastern European and Western Countries in terms of knowledge and implementation of LIFE Nature projects [58], and enable countries with high biodiversity but low project implementation capacity to mobilize organizational and financial resources for achieving EU Biodiversity 2020 goals [6].

## Supporting Information

S1 Dataset Raw data used for performing the network analyses. (XLSX)

S2 Dataset Structural and within–node ERGMs results for the six LIFE Nature partnership networks. (XLSX)

S1 Fig Summary of the methodology. (TIF)

S2 Fig LIFE Nature partnership network for United Kingdom, Netherlands, Portugal and Greece (size of nodes for organisation = degree; circles: red—public authority, blue–NGO, green—park reserve authority, purple—research and education, orange–enterprises, grey–project; dark grey arrow—link to a beneficiary, grey arrow—link to a partner; isolated projects are presented in the left bottom of a network; abbreviations are shown in S4 Table). (TIF)

S1 Table Glossary. (DOCX)

S2 Table Interpretation of ERGM configurations. (DOCX)

S3 Table Country level centrality metrics. (DOCX)

S4 Table Abbreviation labels of each organisation by country. (DOCX)
