# Supplementary figures and images for: Collaboration Networks in Applied Conservation Projects across Europe
Source: PLoS One. 2016 Oct 10;11(10):e0164503. doi: 10.1371/journal.pone.0164503 (PMC5056702; doi:10.1371/journal.pone.0164503)

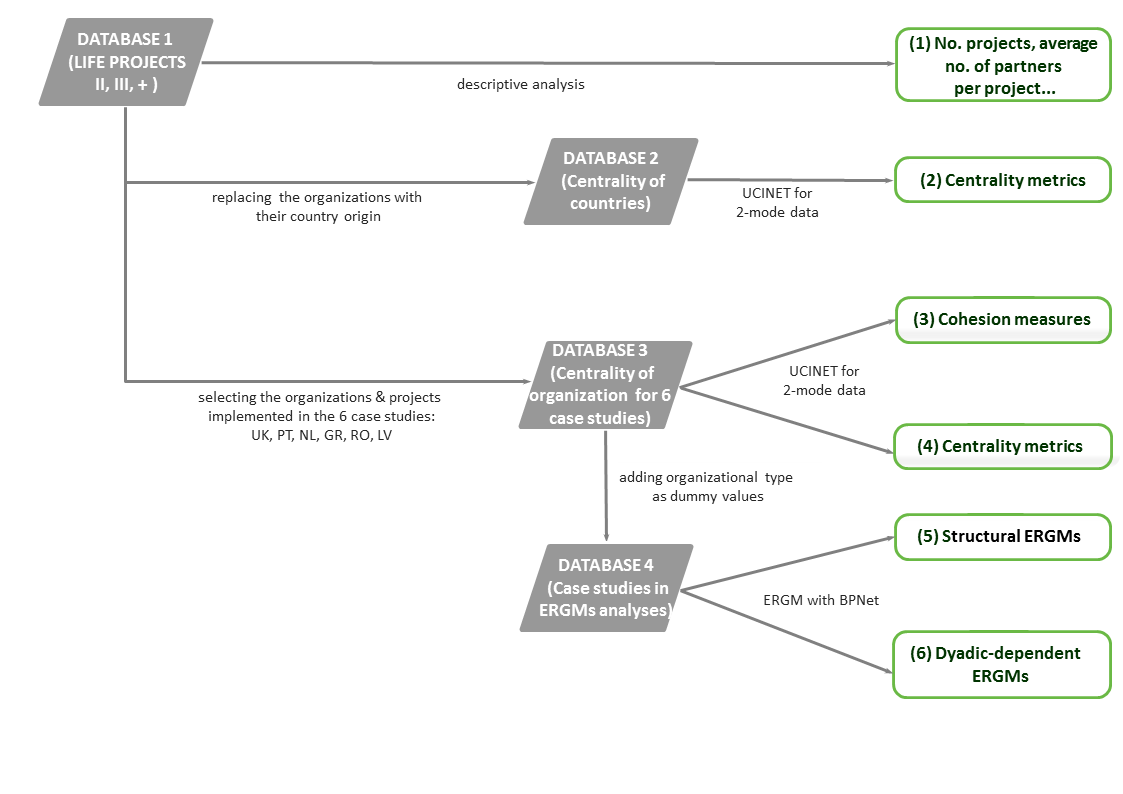

Supplement: S1 Fig — (TIF) [file pone.0164503.s003.tif]

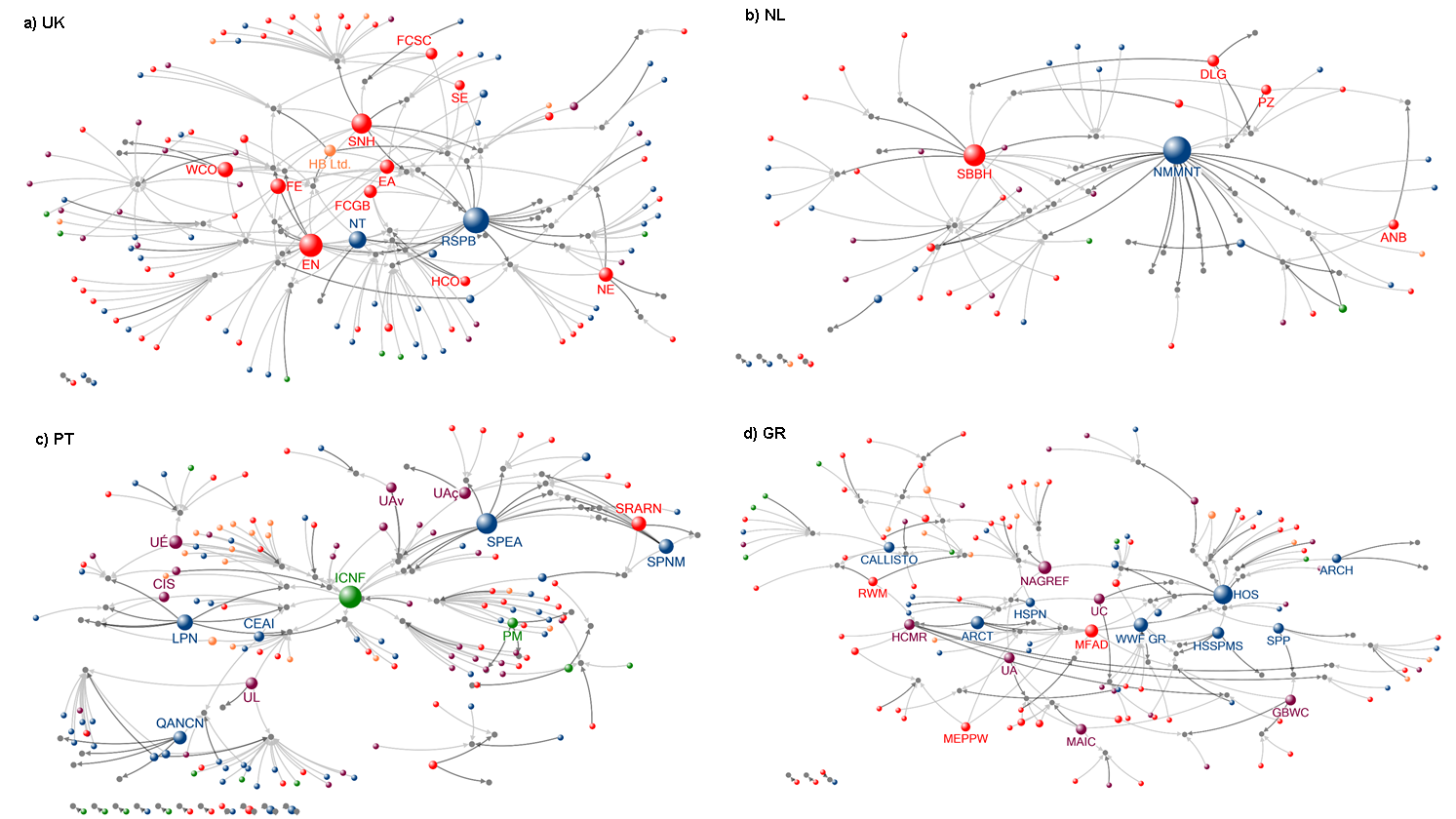

Supplement: S2 Fig — (TIF) [file pone.0164503.s004.tif]
