# Supplementary material for: Collaboration Networks in Applied Conservation Projects across Europe
Source: PLoS One. 2016 Oct 10;11(10):e0164503. doi: 10.1371/journal.pone.0164503 (PMC5056702; doi:10.1371/journal.pone.0164503)
Supplement: S1 Table — (DOCX) [file pone.0164503.s005.docx]

**S1.Table. Glossary:**

**Social network** - A finite set (or sets) of actors (organisations) and the connections (partnerships) between them.

**Two-mode network** - A 2-dimensional matrix containing 2 different sets of entities [38] i.e., organizations and projects.

**Graph** - A common representation of social networks, consisting of two dimensions: actors and relations (also called nodes and edges).

**Node** - Entity in a graph, element of a network (also called vector) [38].

**Edge** - Relationship between nodes (e.g., if we consider a project partnership as a graph, every partner is connected to the project with an edge).

**Short-path** - minimum number of ties linking the two nodes [24].

**ERGMs** - Exponential random graph models represent statistical models for analysing data about social networks [39].

**Network diameter** - Average minimum distance between pairs of nodes.

**Degree of fragmentation -** ratio between the number of pairs of nodes that are not connected in the fragmented network to the possible number of pairs in the original fully connected network [21].

**Density** - The number of ties in the network, expressed as a proportion of the total possible number of ties [36].

**Geodesic distance** - The length of the shortest path between two nodes [36].

**Centrality** - A property of a node’s position in a network [36].

**Degree** - The degree centrality of a node is defined as the number of edges incident upon that

node [38].

**Eigenvector centrality** - Weighted degree measure in which the centrality of a node is proportional to the sum of centralities of the nodes it is adjacent to [38].

**Betweenness centrality** - the number of geodesic paths that pass through a given node, weighted inversely by the total number of equivalent paths between the same two nodes, including those that do not pass through the given node [38].

**Closeness centrality** - Sum of geodesic distances from a given node to all others [36].
