# Supplementary material for: Collaboration Networks in Applied Conservation Projects across Europe
Source: PLoS One. 2016 Oct 10;11(10):e0164503. doi: 10.1371/journal.pone.0164503 (PMC5056702; doi:10.1371/journal.pone.0164503)
Supplement: S2 Table — (DOCX) [file pone.0164503.s006.docx]

**S2 Table. Interpretation of ERGM configurations**

a) Structural configuration (circles = organizations, squares = projects)

| **Parameter** | **Graph configuration** | **BP Net code** | **Interpretation** |
| --- | --- | --- | --- |
| k-stars |  | SA2 | Network activity (variance in degree distribution). Tendency for projects to include more partners (SA2, SA3). Propensity of organizations to be more active, i.e. to participate in more projects (SP2, SP3). |
|  |  | SP2 |  |
|  |  | SA3 |  |
|  |  | SP3 |  |
| Alternating-stars |  | KSA | Dispersion of projects / organisations degree distribution. Centralization in projects (KSP) and organisations (KSA) degree distribution.  Popular project / organization has an increased probability of receiving further ties. |
|  |  | KSP |  |
| Three-path |  | L3 | Potential closure. Tendency for organisations to participate in popular projects. |
| Four-cycle |  | C4 | Full bipartite closure. Tendency for participating in conservation clusters (given that an organization already collaborates in a project with another organization, there is a tendency to collaborate in a second project with that organization). |

| **Parameter** | **Graph configuration** | **BP Net code** | **Interpretation** |
| --- | --- | --- | --- |
| Alternating-two-paths |   … | KCA | Dispersion of affiliation sharing distribution. Tendency for organizations to share multiple projects (KCA) and for projects to share multiple organizations (KCP). |
|  |   … | KCP |  |
| Edge-cycles |  | ECA | Interaction between closure and activity. Organizations (ECA) or projects (ECP) hubs tendency to be involved in closed structures. Hubs are nodes with degree *h*+2, where h is the number of nodes liked by four-cycle structure (in our graph configuration *h*=1). |
|  |  | ECP |  |
| Alternating-edge-cycles |   … | AECA | Centralization in activity associated with closure. A greater centralization for degree distribution of organizations (AECA) or projects (AECP) that are part of a closure structure. |
|  |     … | AECP |  |

b). Binary attribute effects. (circles = organizations with attribute, squares = projects, empty circles or squares = elements with or without attributes)

| **Parameter** | **Graph configuration** | **BPNet code** | **Interpretation** |
| --- | --- | --- | --- |
| Organizational type density |  | *Attr*-RA | Tendency for an organizational type (with the respective attribute) to form ties, regardless of the attributes of the linked node. |
| Across-type bridging for organizations |  | *Attr*-TsoA1 | The tendency for the respective organizational type to connect to projects implemented by different organizational types. |
| Within-type bridging for organizations |  | *attr*TsoA2 | The tendency for an organizational type to connect to projects involving same types of organizations. |
