# Supplementary material for: Collaboration Networks in Applied Conservation Projects across Europe
Source: PLoS One. 2016 Oct 10;11(10):e0164503. doi: 10.1371/journal.pone.0164503 (PMC5056702; doi:10.1371/journal.pone.0164503)
Supplement: S3 Table — (DOCX) [file pone.0164503.s007.docx]

**S3 Table. Country level centrality metrics**

| **Country** | **Degree** | **Eigenvector** | **Closeness** | **Betweenness** | **Label** |
| --- | --- | --- | --- | --- | --- |
| Italy | 0.177 | 0.989 | 0.299 | 0.329 | IT |
| Spain | 0.149 | 0.129 | 0.316 | 0.278 | ES |
| Germany | 0.093 | 0.006 | 0.313 | 0.225 | DE |
| France | 0.071 | 0.044 | 0.299 | 0.156 | FR |
| Greece | 0.05 | 0.033 | 0.296 | 0.13 | EE |
| United Kingdom | 0.051 | 0.005 | 0.291 | 0.113 | UK |
| Netherlands | 0.041 | 0.008 | 0.318 | 0.11 | NL |
| Portugal | 0.053 | 0.023 | 0.295 | 0.096 | PT |
| Belgium | 0.051 | 0.007 | 0.253 | 0.091 | BE |
| Finland | 0.044 | 0.001 | 0.248 | 0.084 | FI |
| Austria | 0.038 | 0.006 | 0.249 | 0.073 | AT |
| Denmark | 0.03 | 0.001 | 0.275 | 0.067 | DK |
| Romania | 0.038 | 0.006 | 0.243 | 0.067 | RO |
| Hungary | 0.033 | 0.001 | 0.249 | 0.066 | HU |
| Sweden | 0.038 | 0 | 0.218 | 0.066 | SE |
| Poland | 0.035 | 0.001 | 0.271 | 0.065 | PL |
| Bulgaria | 0.017 | 0.017 | 0.285 | 0.057 | BG |
| Slovakia | 0.018 | 0 | 0.225 | 0.042 | SK |
| Latvia | 0.022 | 0 | 0.24 | 0.035 | LV |
| Slovenia | 0.018 | 0.02 | 0.236 | 0.03 | SI |
| Estonia | 0.017 | 0 | 0.239 | 0.025 | EE |
| Ireland | 0.013 | 0.001 | 0.257 | 0.023 | IRL |
| Lithuania | 0.011 | 0 | 0.218 | 0.014 | LT |
| Luxembourg | 0.008 | 0 | 0.214 | 0.013 | LU |
| Norway | 0.002 | 0.001 | 0.278 | 0.01 | NO |
| Cyprus | 0.007 | 0 | 0.206 | 0.008 | CY |
| Czech Republic | 0.005 | 0 | 0.157 | 0.008 | CZ |
| Malta | 0.004 | 0 | 0.201 | 0.005 | MT |
| Croatia | 0.002 | 0.01 | 0.249 | 0.004 | HR |
| Russia | 0.001 | 0 | 0.208 | 0 | RU |
| Iceland | 0.001 | 0 | 0.185 | 0 | IS |
