# Supplementary material for: Collaboration Networks in Applied Conservation Projects across Europe
Source: PLoS One. 2016 Oct 10;11(10):e0164503. doi: 10.1371/journal.pone.0164503 (PMC5056702; doi:10.1371/journal.pone.0164503)
Supplement: S4 Table — (DOCX) [file pone.0164503.s008.docx]

**S4 Table. Abbreviation labels of each organisation by country**

1. **United Kingdom**

| **Organisations UK** | |
| --- | --- |
| **Name of the organisation** | **Label** |
| Royal Society for the Protection of Birds (RSPB), UK | RSPB |
| English Nature, UK | EN |
| Scottish Natural Heritage, UK | SNH |
| National Trust, UK | NT |
| Forest Enterprise, UK | FE |
| Countryside Council for Wales, UK | WCO |
| Environment Agency, UK | EA |
| Natural England, UK | NE |
| Forestry Commission, UK | FCGB |
| Forestry Commission Scotland, UK | FCSC |
| Caledonian Partnership, Highland Birchwoods Ltd., UK | HB Ltd. |
| Scottish Executive, UK | SE |
| Hampshire County Council, UK | HCO |

1. **Netherlands**

| **Organisations NL** | |
| --- | --- |
| **Name of the organisation** | **Label** |
| Natuurmonumenten, NL | NMMNT |
| Staatbosbeheer, NL | SBBH |
| Dienst Landelijk Gebied, NL | DLG |
| Agentschap voor Natuur en Bos, NL | ANB |
| Provincie Zeeland, NL | PZ |

1. **Portugal**

| **Organisations PT** | |
| --- | --- |
| **Name of the organisation** | **Label** |
| ICNF (Instituto da Conservação da Natureza e das Florestas), PT | ICNF |
| Sociedade Portuguesa para a Estudo das Aves, PT | SPEA |
| Liga para a Protecção da Natureza, PT | LPN |
| Serviço do Parque Natural da Madeira, PT | SPNM |
| Direcção Regional de Florestas Secretaria Regional do Ambiente e Recursos Naturais, PT | SRARN |
| Universidade de Évora, PT | *UÉ* |
| QUERCUS Associação Nacional de Conservação da Natureza, PT | QANCN |
| Universidade dos Açores, PT | UAç |
| Universidade de Lisboa, PT | UL |
| Universidade de Aveiro, PT | UAv |
| Centro de Investigação e de Intervenção Social (CIS), PT | CIS |
| Centro de Estudos da Avifauna Ibérica, PT | CEAI |
| Parque Natural da Madeira, PT | PM |

1. **Greece**

| **Organisations GR** | |
| --- | --- |
| **Name of the organisation** | **Label** |
| Hellenic Ornithological Society, GR | HOS |
| WWF, GR | WWF GR |
| Civil Society for the Protection of Natural Environment and Wildlife (Arcturos), GR | ARCT |
| National Agricultural Research Foundation/Forest Research Institute (NAGREF/FRI), GR | NAGREF |
| The Ministry of Food and Agriculture Development, GR | MFAD |
| Hellenic Society for the Study and Protection of the Monk Seal, GR | HSSPMS |
| CALLISTO Wildlife and Nature Conservation Society, GR | CALLISTO |
| Hellenic Centre for Marine Research Institute of Inland Waters, GR | HCMR |
| Mediterranean Agronomic Institute of Chania, GR | MAIC |
| Society for the protection of Prespa, GR | SPP |
| The Goulandris Natural History Museum/Greek Biotope Wetland Centre, GR | GBWC |
| University of Athens, GR | UA |
| University of Crete Natural History Museum of Crete, GR | UC |
| Archelon/The Sea Turtle Protection Society of Greece, GR | ARCH |
| Hellenic Society for the Protection of Nature, GR | HSPN |
| Ministry of Environment, Physical Planning and Public Works, GR | MEPPW |
| Region of Western Macedonia, GR | RWM |

1. **Romania**

| **Organisations RO** | |
| --- | --- |
| **Name of the organisation** | **Label** |
| Romanian Ornithological Society, RO | SOR |
| Bucharest University, RO | UB |
| Forest Research and Management Institute (ICAS), RO | ICAS |
| Milvus Group Association, RO | MLVS |
| Romanian Academy, RO | ROAC |
| Kiskunság National Park Directorate, HU | KNPHU |
| Ministry of Environment, RO | MENV |
| MME/BirdLife, HU | MMEHU |
| Timisoara Environment Protection Agency, RO | EPATM |
| Vrancea Environment Protection Agency, RO | EPAVN |
| BNPD(Bükk National Park Directorate), HU | BNPHU |
| Caras Severin Environment Protection Agency, RO | EPACS |
| Explorers Speleological Association (ASER), RO | ASER |
| National Forest Authority ROMSILVA, RO | RMSLV |
| National Museum of Natural History Grigore Antipa, RO | NMNH |
| Transilvania University of Brașov, RO | UTB |
| WWF, RO | WWF RO |

1. **Latvia**

| **Organisations LV** | |
| --- | --- |
| **Name of the organisation** | **Label** |
| Latvian Fund for Nature, LV | LVFN |
| Nature Conservation Agency, LV | NCAG |
| Latvian Ornithological Society, LV | LVOS |
| Association of Documentary Film and Photography ELM MEDIA, LV | ELMM |
| State Joint Stock Company “Latvian State Forests”, LV | SJSCLSF |
| University of Latvia, LV | UL |
| Institute for Environmental Solutions (IES), LV | IES |
| National Armed Forces, LV | NAF |
